# MMP-2 geno-phenotype is prognostic for colorectal cancer survival, whereas MMP-9 is not

**DOI:** 10.1038/sj.bjc.6604380

**Published:** 2008-05-27

**Authors:** A M J Langers, C F M Sier, L J A C Hawinkels, F J G M Kubben, W van Duijn, J J van der Reijden, C B H W Lamers, D W Hommes, H W Verspaget

**Affiliations:** 1 Department of Gastroenterology–Hepatology, Leiden University Medical Centre, Leiden, The Netherlands

**Keywords:** metalloproteinase, SNP, survival, prognosis

## Abstract

The prognostic significance of single-nucleotide polymorphisms (SNPs) and tumour protein levels of MMP-2 and MMP-9 was evaluated in 215 colorectal cancer patients. Single-nucleotide polymorphism MMP-2_−1306T_ and high MMP-2 levels were significantly associated with worse survival. Extreme tumour MMP-9 levels were associated with poor prognosis but SNP MMP-9_−1562C>T_ was not. Tumour MMP levels were not determined by their SNP genotypes.

Enhanced tumour matrix metalloproteinase (MMP) levels have been correlated to decreased patients' survival in various types of cancer (Sienel *et al*, 2003; Pellikainen *et al*, 2004; Kubben *et al*, 2006b; Hilska *et al*, 2007; de Vicente *et al*, 2007). Next to sheer degraders of extracellular matrix, MMPs are presently regarded as general molecular switches in the microenvironment (Overall and Blobel, 2007). For instance, MMPs function as major regulators of tumour growth by catalysing the release or activation of growth factors, activation or shedding of membrane receptors, or cleavage of matrix/membrane-bound substrates involved in cell proliferation (Cauwe *et al*, 2007). A subgroup of MMPs, the gelatinases (MMP-2 and MMP-9), have been particularly implicated in progression, angiogenesis and metastasis of various cancer types (Turpeenniemi-Hujanen, 2005). In the present study, we evaluated the association between, and the prognostic relevance of tissue protein levels of MMP-2 and MMP-9 and their gene promoter single-nucleotide polymorphisms (SNPs) in a cohort of 215 Dutch colorectal cancer patients.

## MATERIALS AND METHODS

### Patients and study design

Representative, nonnecrotic samples of cancer tissue were collected from 215 patients with colorectal cancer, operated in the Leiden University Medical Centre (December 1983–September 1991). Tissues were snap-frozen and stored at −70°C. Clinical data and follow-up were available for at least 10 years. Macroscopic and microscopic parameters were obtained from the pathology reports. The study was performed according to the instructions and guidelines of the LUMC Medical Ethics Committee.

### Tissue preparation and protein concentration

Tissues were homogenised in 0.1 M. Tris-HCL (pH 7.5) with 0.1% (v/v) Tween 80 buffer and centrifuged twice all at 4°C, as described before (Sier *et al*, 1991), the protein concentrations were determined (Lowry *et al*, 1951) and the supernatants were aliquoted and stored at −70°C. Storage-induced degradation of MMP-2 and MMP-9 was checked by western blots and gelatin zymography prior to ELISA measurements (Sier *et al*, 1996).

### Determination of MMP-2 and MMP-9 in tissue homogenates

The MMP-2 and MMP-9 levels were determined by previously described ELISAs (Gao *et al*, 2005). Polyclonal anti-MMP-2 or monoclonal anti-MMP-9 antibodies were used as catching antibody, incubated with appropriately diluted samples (o/n, 4°C), and polyclonal anti-MMP-2/biotin-labelled goat anti-rabbit-IgG and biotin-labelled polyclonal anti-MMP-9 antibodies for immune-detection combined with avidin-peroxidase and the 3,3′,5,5′-tetramethyl benzidine/H_2_O_2_ substrate solution, and the absorption was read at 450 nm. Sample MMP concentrations were calculated from standard curves and expressed in ng per mg protein.

### Single-nucleotide polymorphism analysis

Tissue DNA was isolated using the salting out method (Miller *et al*, 1988). Single-nucleotide polymorphism (SNP) analysis for MMP-2_−1306C>T_ and MMP-9_−1562C>T_ was performed by tetra-primer ARMS PCR, involving four oligonucleotide primers but no restriction enzymes, or RFLP-PCR, as described earlier (Meijer *et al*, 2006; Kubben *et al*, 2006a).

### Statistical analysis

Statistical analyses were performed using SPSS 12.0 Statistical Package (2004; SPSS Inc., Chicago, IL, USA). Expression differences between groups were calculated using the Mann–Whitney's *U*-test. Log-rank statistics was used for optimal cutoff point analysis. Hardy–Weinberg's analysis was performed using *χ*^2^ or Fisher's exact test to examine differences in the distribution of alleles and genotypes. Correlations between parameters were according to Pearson or Spearman, where appropriate. Overall survival curves were according to Kaplan and Meier. Univariate and multivariate survival analyses were performed using the Cox's proportional hazards method. *P*-values ⩽0.05 were considered significant.

## RESULTS

**Figure 1:**
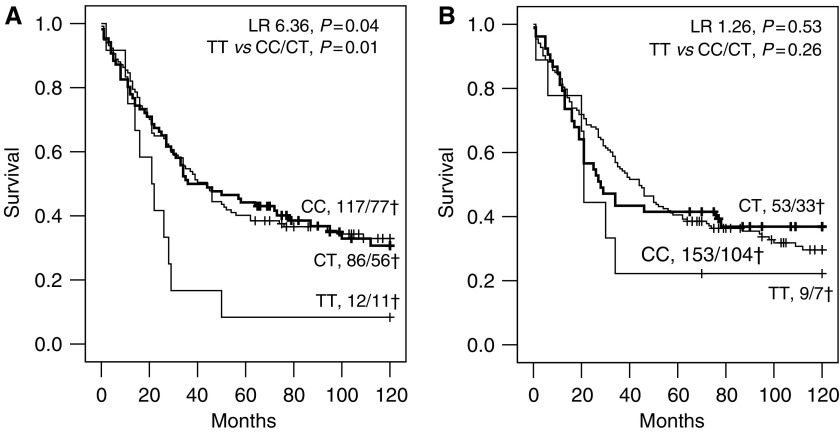
Kaplan–Meier's 10-year survival curves of 215 colorectal cancer patients grouped by their genotype for SNP MMP-2_−1306C>T_ (**A**) and MMP-9_−1562C>T_ (**B**). The total number of patients and the deceased patients (†) are indicated per subgroup.

The genotype distributions of the SNPs for MMP-2 and MMP-9 in 215 colorectal cancer patients are shown in Table 1. The distribution of the polymorphisms in the patients was according to the predicted Hardy–Weinberg's distribution. Both SNPs showed a weak but significant association with TNM stage (Table 1) and MMP-2_−1306C>T_ also with survival (Figure 1A). All other clinicopathological parameters did not show an association with either SNP MMP-2_−1306C>T_ or MMP-9_−1562C>T_, and the latter was also not associated with survival (Figure 1B).

**Figure 2:**
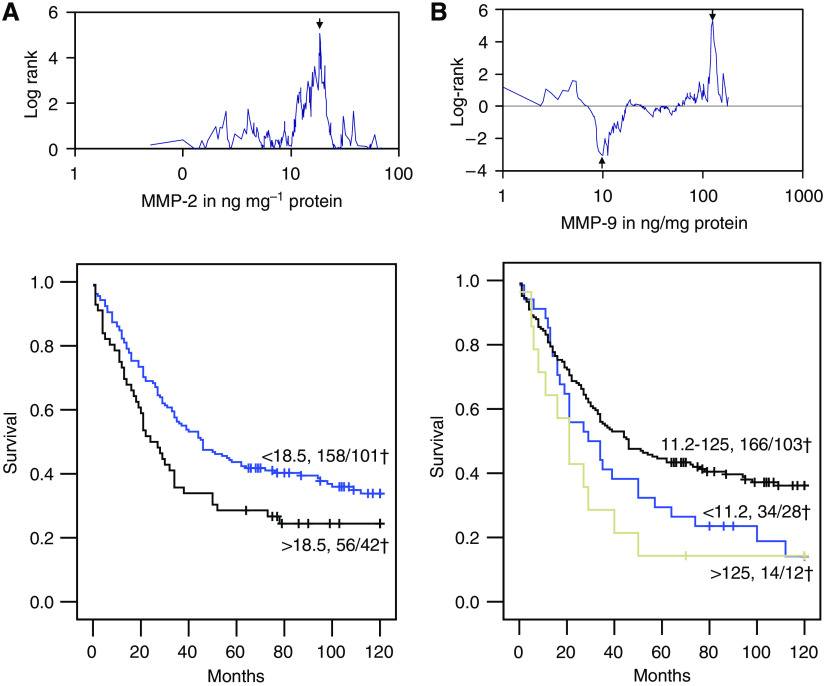
Optimal cutoff point analysis and corresponding Kaplan–Meier's 10-year survival curves for MMP-2 (**A**) and MMP-9 (**B**) in tumour tissue homogenates from 215 colorectal cancer patients. The optimal cutoff points are indicated with arrows. In the survival curves, the total number of patients and the deceased patients (†) are indicated per subgroup.

The protein levels of MMP-2 (median 10.6; range: 0.0–76.6 ng per mg protein) and MMP-9 (median 37.3; range: 0.5–201.9 ng per mg protein) in the colorectal carcinomas were found not to be related to any of the clinicopathological parameters, although for MMP-2 a stepwise increase with TNM stage was discernable (not shown). For the survival analysis, an optimised cutoff point value was identified for the tumour MMP-2 level (18.5 ng per mg protein, LR 5.07, *P*=0.024, Figure 2A). The same approach for MMP-9 resulted in two differently oriented cutoff points; a low MMP-9 value (11.2 ng per mg protein, LR 9.18, *P*=0.010) and a high value (125.0 ng per mg protein, LR 5.31, *P*=0.021), both associated with poor prognosis (Figure 2B).

The MMP-2 and MMP-9 protein levels in the colorectal cancer homogenates did not correlate with their respective SNP genotypes. The median values for MMP-2_−1306C>T_ were 10.9, 9.5 and 11.6 ng mg^−1^ for genotypes CC, CT and TT, respectively. Even an apparent enhancement of MMP-9 protein associated with the TT genotype of SNP MMP-9_−1562C>T_ in tumours did not reach significance (CC/CT *vs* TT, medians 36.5/26.3 *vs* 44.4 ng mg^−1^, *P*=0.28).

Univariate Cox's survival analyses confirmed the association of the MMP-2 SNP and the protein levels of MMP-2 and MMP-9 with survival (Table 2). Multivariate analysis against the prognosis-associated parameters gender, age and TNM classification showed that the MMP-2 SNP was independently associated with survival, whereas the tumour protein levels of MMP-2 just lost and MMP-9 completely lost their significance.

## DISCUSSION

MMP-2 and MMP-9 are proteinases implicated in cancer progression. We showed previously that high tumour levels of MMP-2 in gastric carcinomas were consistently associated with a worse survival (Sier *et al*, 1996; Kubben *et al*, 2006b). For enhanced MMP-9 levels, the relation with survival was more ambivalent (Kubben *et al*, 2006b, 2007). Also in our present cohort of colorectal carcinoma patients, we found that MMP-2 and MMP-9 levels within the tumours are of significance to survival. The cutoff point analysis showed a broad range of MMP-2 levels with a significant and unidirectional relation with survival outcome: high tumour MMP-2 levels are unfavourable for the patients' prognosis. Our ELISA-derived MMP-2 data correspond very well with a recent immunohistochemical study in a group of 351 colorectal cancer patients, showing that high expression of MMP-2 in malignant epithelium as well as in the surrounding stroma was associated with reduced survival (Hilska *et al*, 2007). Similar analysis on our tumour MMP-9 data revealed that not only patients with the highest, but also those with the lowest levels had a worse survival than patients with intermediate levels. Duality of tumour MMP-9 levels with respect to survival has been recognised before in immunohistochemistry-based studies. For example, extensive MMP-9 staining in ovarian cancer cells was associated with a longer survival, as opposed to a shorter survival with a higher stromal expression of MMP-9 (Sillanpaa *et al*, 2007). Earlier morphometric studies indicated that the degree of MMP-9 expression in tumour-associated lymphocytes, macrophages and neutrophils was inversely associated with invasion and metastasis in colorectal cancer (Takeha *et al*, 1997). These observations are relevant to our findings because a low level of MMP-9 in the tumour homogenates might indicate a lack of infiltration of the tumours with MMP-9 containing leukocytes, known to exert anticancer effects (Nielsen *et al*, 1996; Owen *et al*, 2004), leading to an adverse prognosis. In addition, MMP-9-mediated cleavage of extracellular matrix components is also known to generate antiangiogenesis inhibitors like angiostatin (Patterson and Sang, 1997). Low MMP-9 expression in the tumour, leading to insufficient production of antiangiogenic factors, could also contribute to the worse prognosis of these patients (Pozzi *et al*, 2002). However, MMP-9 has a wide range of substrates, including various growth factors and several types of collagen, which after cleavage contribute to the process of invasion, angiogenesis and metastasis of tumours, explaining why high tumour levels of MMP-9, in general, are associated with a poor prognosis.

Differences in expression of metalloproteinases might, in part, be explained by genotypic variation. Because the investigated SNPs of MMP-2 and MMP-9 are located in the promoter region of the gene, a correlation between polymorphism and protein expression might be expected. The C → T transition at the −1306 position of the MMP-2 gene promoter prevents binding of the stimulating Sp1 transcription factor, whereas a change at the −1562 position of the MMP-9 gene decreases binding of a repression factor (Zhang *et al*, 1999; Price *et al*, 2001). *In vitro* MMP-2 expression levels by colon cancer cell lines containing the CC genotype were indeed higher compared with cells with the CT genotype (Xu *et al*, 2004). In our cohort of patients, however, we found no relationship between MMP-2 and MMP-9 polymorphisms and the tumour protein levels, likely caused by the complicated regulatory posttranslational mechanisms for proteinases in multicellular tumour tissues. Nevertheless, we did find significant associations between MMP-2_−1306C>T_ and TNM stage and survival, that is, worse prognosis in patients with the TT genotype. This association with the outcome of the patients support the recent confirmation of MMP-2 as one of the candidate cancer genes (CAN genes) by the number and nature of mutations and pathways in colorectal cancer (Sjöblom *et al*, 2006). Our results for SNP MMP-9_−1562C>T_ correspond well with data from Asiatic patients (Xu *et al*, 2007), indicating that this polymorphism is not directly involved in the process of colorectal carcinogenesis.

Summarising, we showed that MMP-9, mechanistically probably the most interesting of the gelatinases, is not a likely candidate as a simple prognostic indicator. Despite the absence of a correlation between promoter-located SNP −1306C>T in the MMP-2 gene with tumour MMP-2 levels, both parameters were significantly associated with survival, indicating MMP-2 as a consistent independent prognostic factor in colorectal cancer.

## Figures and Tables

**Table 1:** Genotype distribution of single-nucleotide polymorphisms (SNPs) for MMP-2 and MMP-9 in 215 colorectal carcinoma patients compared with the expected Hardy–Weinberg distribution (H-W)

**SNP**		**All**	TNM stage*			
**genotype**	**H-W**	**patients**	**1**	**2**	**3**	**4**
*MMP-2*_*−1306C>T*_	(%)	% *(N)*	% *(N)*	% *(N)*	% *(N)*	% *(N)*
CC	55.4	54.4 (117)	69.4 (25)	57.1 (48)	52.2 (35)	32.1 (9)
CT	38.1	40.0 (86)	27.8 (10)	40.5 (34)	41.8 (28)	50.0 (14)
TT	6.6	5.6 (12)	2.8 (1)	2.4 (2)	6.0 (4)	17.9 (5)
						
*MMP-9*_*−1562C>T*_						
CC	69.7	71.2 (153)	83.3 (30)	70.2 (59)	73.1 (49)	53.6 (15)
CT	27.6	24.7 (53)	11.1 (4)	28.6 (24)	23.9 (16)	32.1 (9)
TT	2.7	4.1 (9)	5.6 (2)	1.2 (1)	3.0 (2)	14.3 (4)

MMP=matrix metalloproteinase

^*^χ^2^values for TNM stage distribution of MMP-2 and MMP-9 were, respectively, 15.9 (*P*=0.01) and 14.9 (*P*=0.02).

**Table 2:** Univariate and multivariate Cox's proportional hazard 10-year survival analysis of 215 colorectal cancer patients

			**Univariate**			**Multivariate**		
		***n***	**HR**	**CI 95%**	***P***	**HR**	**CI 95%**	***P***
*Parameter*								
Gender	F *vs* M	91–124	1.363	0.973–0908	0.071	1.362	0.973–1.908	0.072
Age	<65 years >	73–142	2.123	1.448–3.113	**0.000**	2.390	1.625–3.516	**0.000**
TNM	1/2 *vs* 3/4	120–95	2.316	1.662–3.228	**0.000**	2.548	1.822–3.564	**0.000**
Localisation	Right *vs* rest	75–140	1.023	0.726–1.442	0.896		Not included	
Diameter	<5 cm >	92–123	1.310	0.936–1.834	0.116		Not included	
Differentiation	Good *vs* rest	38–174	1.129	0.789–1.614	0.506		Not included	
								
*MMP proteins*								
MMP-2	<18.5 ng mg^−1^>	158–56	1.504	1.048–2.158	**0.027**	1.417	0.982–2.043	**0.062**
MMP-9	11.2–125	166	1.000					
	<11.2	34	1.526	1.004–2.319	**0.048**	0.787	0.516–1.201	0.266
	>125	14	2.135	1.171–3.895	**0.013**	1.109	0.561–2.189	0.766
								
*MMP SNPs*								
MMP-2	CC/CT *vs* TT	203–12	1.471	1.079–2.006	**0.015**	1.395	1.019–1.911	**0.038**
MMP-9	CC/CT *vs* TT	206–9	1.239	0.847–1.813	0.270	1.114	0.757–1.638	0.584

CI=confidence interval; HR=hazard ratio; MMP=matrix metalloproteinase.

Multivariate analysis was performed by adding every single MMP-related parameter to the dichotomised, prognosis-associated clinicopathological parameters gender, age and TNM stage. Entries in bold indicate significant, or in case of MMP-2 almost significant *P*-values.
